# Factors Associated With Relapse and Treatment of Myelin Oligodendrocyte Glycoprotein Antibody–Associated Disease in the United Kingdom

**DOI:** 10.1001/jamanetworkopen.2021.42780

**Published:** 2022-01-10

**Authors:** Chanjira Satukijchai, Romina Mariano, Silvia Messina, Mario Sa, Mark R. Woodhall, Neil P. Robertson, Lim Ming, Evangeline Wassmer, Rachel Kneen, Saif Huda, Anu Jacob, Camilla Blain, Christopher Halfpenny, Cheryl Hemingway, Eoin O'Sullivan, Jeremy Hobart, Leonora K. Fisniku, Roswell Martin, Ruth Dopson, Sarah A. Cooper, Victoria Williams, Patrick J. Waters, Sithara Ramdas, Maria Isabel Leite, Jacqueline Palace

**Affiliations:** 1 Nuffield Department of Clinical Neurosciences, John Radcliffe Hospital, University of Oxford, Oxford, United Kingdom; 2 Neuroscience Center, Bangkok International Hospital, Bangkok, Thailand; 3 Division of Neurology, Department of Medicine, Faculty of Medicine, Siriraj Hospital, Mahidol University, Bangkok, Thailand; 4 Department of Clinical Neurology, John Radcliffe Hospital, Oxford University Hospitals Trust, Oxford, United Kingdom; 5 Paediatric Neurology, Great Ormond Street Hospital for Children, London, United Kingdom; 6 Department of Neurology, Division of Psychological Medicine and Clinical Neuroscience, Cardiff University, University Hospital of Wales, Cardiff, United Kingdom; 7 Children’s Neurosciences, Evelina London Children’s Hospital at Guy’s and St Thomas’ National Health Service Foundation Trust, London, United Kingdom; 8 Department of Women and Children’s Health, School of Life Course Sciences, King’s College London, United Kingdom; 9 Birmingham Women’s and Children’s National Health Service Foundation Trust, Birmingham, United Kingdom; 10 School of Life and Health Sciences, Aston University, Birmingham, United Kingdom; 11 Alder Hey Children's National Health Service Foundation Trust, Liverpool, United Kingdom; 12 Department of Neurology, Walton Centre National Health Service Foundation Trust, Liverpool, United Kingdom; 13 Cleveland Clinic Abu Dhabi, Abu Dhabi, United Arab Emirates; 14 St George’s University Hospitals National Health Service Foundation Trust, London, United Kingdom; 15 University Hospitals Southampton National Health Service Foundation Trust, Southampton, United Kingdom; 16 Department of Paediatric Neurology, Great Ormond Street Hospital for Children, London, United Kingdom; 17 Department of Ophthalmology, Kings College Hospital, London, United Kingdom; 18 Plymouth University Peninsula Schools of Medicine and Dentistry, Plymouth, United Kingdom; 19 University Hospitals Plymouth National Health Service Foundation Trust, United Kingdom; 20 University Hospitals Sussex National Health Service Foundation Trust, Brighton, United Kingdom; 21 Brighton and Sussex Medical School, Brighton, United Kingdom; 22 Gloucestershire Hospitals National Health Service Foundation Trust, Gloucestershire, United Kingdom; 23 Preventive Neurology Unit, Wolfson Institute of Preventive Medicine, Queen Mary University London, London, United Kingdom; 24 Royal London Hospital, Barts Health National Health Service Foundation Trust, United Kingdom; 25 Guy’s and St Thomas’ National Health Service Foundation Trust, London, United Kingdom; 26 Department of Paediatric Neurology, John Radcliffe Hospital, Oxford, United Kingdom

## Abstract

**Importance:**

Longer-term outcomes and risk factors associated with myelin oligodendrocyte glycoprotein antibody–associated disease (MOGAD) are not well established.

**Objective:**

To investigate longer-term risk of relapse and factors associated with this risk among patients with MOGAD.

**Design, Setting, and Participants:**

This large, single-nation, prospective cohort study was conducted among 276 patients with MOGAD at 5 health care centers in the UK. Data from January 1973 to March 2020 were collected from 146 patients at Oxford and its outreach sites, 65 patients at Liverpool, 32 patients at a children’s hospital in Birmingham, 22 patients at a children’s hospital in London, and 11 patients at Cardiff, Wales. Data were analyzed from April through July 2020.

**Main Outcomes and Measures:**

Risk of relapse and annualized relapse rate were evaluated according to different baseline features, including onset age, onset phenotype, and incident vs nonincident group, with the incident group defined as patients diagnosed with antibodies against myelin oligodendrocyte glycoprotein before a second attack. Time to next relapse among patients experiencing relapse was measured and compared between the maintenance therapy subgroup and each first-line treatment group. The no-treatment group was defined as the off-treatment phase among patients who were relapsing, which could occur between any attack or between the last attack and last follow-up.

**Results:**

Among 276 patients with MOGAD, 183 patients were identified as being part of the incident group. There were no differences in mean (SD) onset age between total and incident groups (26.4 [17.6] years vs 28.2 [18.1] years), and female patients were predominant in both groups (166 [60.1%] female patients vs 106 [57.9%] female patients). The most common presentation overall was optic neuritis (ON) (119 patients among 275 patients with presentation data [43.3%]), while acute disseminated encephalomyelitis (ADEM), brain, or brainstem onset was predominant among 69 patients aged younger than 12 years (47 patients [68.1%]), including 41 patients with ADEM (59.4%). In the incident group, the 8-year risk of relapse was 36.3% (95% CI, 27.1%-47.5%). ON at onset was associated with increased risk of relapse compared with transverse myelitis at onset (hazard ratio [HR], 2.66; 95% CI, 1.01-6.98; *P* = .047), but there was no statistically significant difference with adjustment for a follow-on course of corticosteroids. Any TM at onset (ie, alone or in combination with other presentations [ie, ON or ADEM, brain, or brain stem]) was associated with decreased risk of relapse compared with no TM (HR, 0.41; 95% CI, 0.20-0.88; *P* = .01). Young adult age (ie, ages >18-40 years) was associated with increased risk of relapse compared with older adult age (ie, ages >40 years) (HR, 2.71; 95% CI, 1.18-6.19; *P* = .02). First-line maintenance therapy was associated with decreased risk of relapse when adjusted for covariates (prednisolone: HR, 0.33; 95% CI, 0.12-0.92; *P* = .03; prednisolone, nonsteroidal immunosuppressant, or combined: HR, 0.51; 95% CI, 0.28-0.92; *P* = .03) compared with the no-treatment group.

**Conclusions and Relevance:**

The findings of this cohort study suggest that onset age and onset phenotype should be considered when assessing subsequent relapse risk and that among patients experiencing relapse, prednisolone, first-line immunosuppression, or a combination of those treatments may be associated with decreased risk of future relapse by approximately 2-fold. These results may contribute to individualized treatment decisions.

## Key Points

**Question:**

Which factors are associated with long-term risk of relapse in myelin oligodendrocyte glycoprotein antibody–associated disease (MOGAD)?

**Findings:**

In this cohort study of 276 patients with MOGAD from 5 UK health care centers, young adults had increased relapse risk, and clinical presentation at onset with any transverse myelitis was associated with decreased relapse risk. Treatment with prednisolone or nonsteroidal immunosuppressant among patients experiencing relapse was associated with a decreased risk of subsequent relapse.

**Meaning:**

This study found that onset age and phenotype at onset were factors associated with risk of relapse among patients with MOGAD and that prednisolone and first-line immunosuppression were associated with decreased risk of subsequent relapse among patients experiencing relapse.

## Teaser

This cohort study investigates factors associated with long-term risk of relapse among patients with myelin oligodendrocyte glycoprotein antibody–associated disease in the United Kingdom.

## Introduction

Myelin oligodendrocyte glycoprotein antibody–associated disease (MOGAD) is an inflammatory demyelinating disease of the central nervous system with antibodies against myelin oligodendrocyte glycoprotein (MOG-Ab) that are expressed on the cell surface of oligodendrocytes^1^ and myelin sheath in the central nervous system. The disease is a recently characterized acquired demyelinating syndrome, which has been shown in several reports^2,3,4,5^ to have clinical and pathological features distinct from multiple sclerosis (MS) and aquaporin-4 antibody–positive neuromyelitis spectrum disorder (AQP4-NMOSD).^6,7,8^

There appears to be an age-related factor associated with the onset phenotype, with adult cohorts having an increased proportion of ON involvement^9,10,11,12^ and pediatric cohorts having acute disseminated encephalomyelitis (ADEM).^13,14,15^ MOGAD is likely to have at least 2-fold as the prevalence of NMOSD in some regions.^16^

Because MOGAD can range from a monophasic condition to a treatment-resistant relapsing disorder, it is essential to understand the risk of long-term relapse and factors associated with prognosis when deciding immunosuppressive treatments.^9,11,17,18,19,20^ However, this risk is unclear owing to the use of nonincident cohorts or short follow-up times in previous studies. Additionally, most previous studies have reported results from adult-predominant centers or pediatric services; thus, the association of age with outcome may differ by study.

We collected data from the 5 main hospitals in the UK that care for patients with MOGAD, including 2 pediatric specialist centers in the UK, to study risk of relapse, factors associated with relapse, and treatment response among UK patients with MOGAD across a range of ages with longer follow-up times.

## Methods

Patients included in this cohort study had been enrolled according to research and development review board with informed consent or service evaluation committee approval for each of the 5 health care centers (ie, National Research Ethics Service Committee London-Hampstead Research Ethics Committee, South East Wales Research Ethics Committee, Birmingham Children’s Hospital and Evelina London Children’s Hospital West Midlands-South Birmingham Research Ethics Committee, and Oxford Research Ethics Committee) with data collected as part of standard care subsequently anonymized, pooled, and analyzed by the coordinating center. This study followed the Strengthening the Reporting of Observational Studies in Epidemiology (STROBE) reporting guideline for cohort studies.

### Study Design and Clinical Data Collection

This UK study of 276 patients with MOGAD used databases of patients from 5 health care centers: 146 patients at Oxford and its outreach sites, 65 patients at Liverpool, 32 patients at a children’s hospital in Birmingham, 22 patients at a children’s hospital in London, and 11 patients at Cardiff, Wales. All patients had clinical events in keeping with MOGAD and were positive on the cell-based IgG1 assay^21,22^ in the Oxford Autoimmune Neurology Diagnostic Laboratory. Shorter-term data (ie, with a last follow-up date of 2016) was previously reported from 75 patients in the Oxford group, which included a small incident group of 44 patients.^11^

Clinical data included sex, self-identified race, age at onset of MOGAD, family history of autoimmune disease, clinical attack phenotype, number of attacks, disease duration, treatment of acute attacks, chronic immunosuppressant therapy, and presence of cerebrospinal fluid oligoclonal bands. Race was collected because of the observed association of race with outcomes in AQP4-NMOSD to investigate if there were similar associations among patients with MOGAD.^6^ Clinical presentation at onset was categorized into 4 clinical subgroups: ON, TM, simultaneous ON with TM, and ADEM, brain, or brainstem. The latter categorization included individuals who presented with a combination of brain or brainstem attacks with other onset locations, such as ON and TM (eTable 1 in the Supplement).

To analyze risk of relapse, we identified from the total cohort an incident group (prospectively followed from onset) defined as individuals diagnosed with MOG antibodies before a second attack. This was done in order to remove the bias of overestimating relapse risk by enriching data with individuals referred years after onset only because they presented with a relapse (ie, retrospectively recruited).

**Figure 1.**
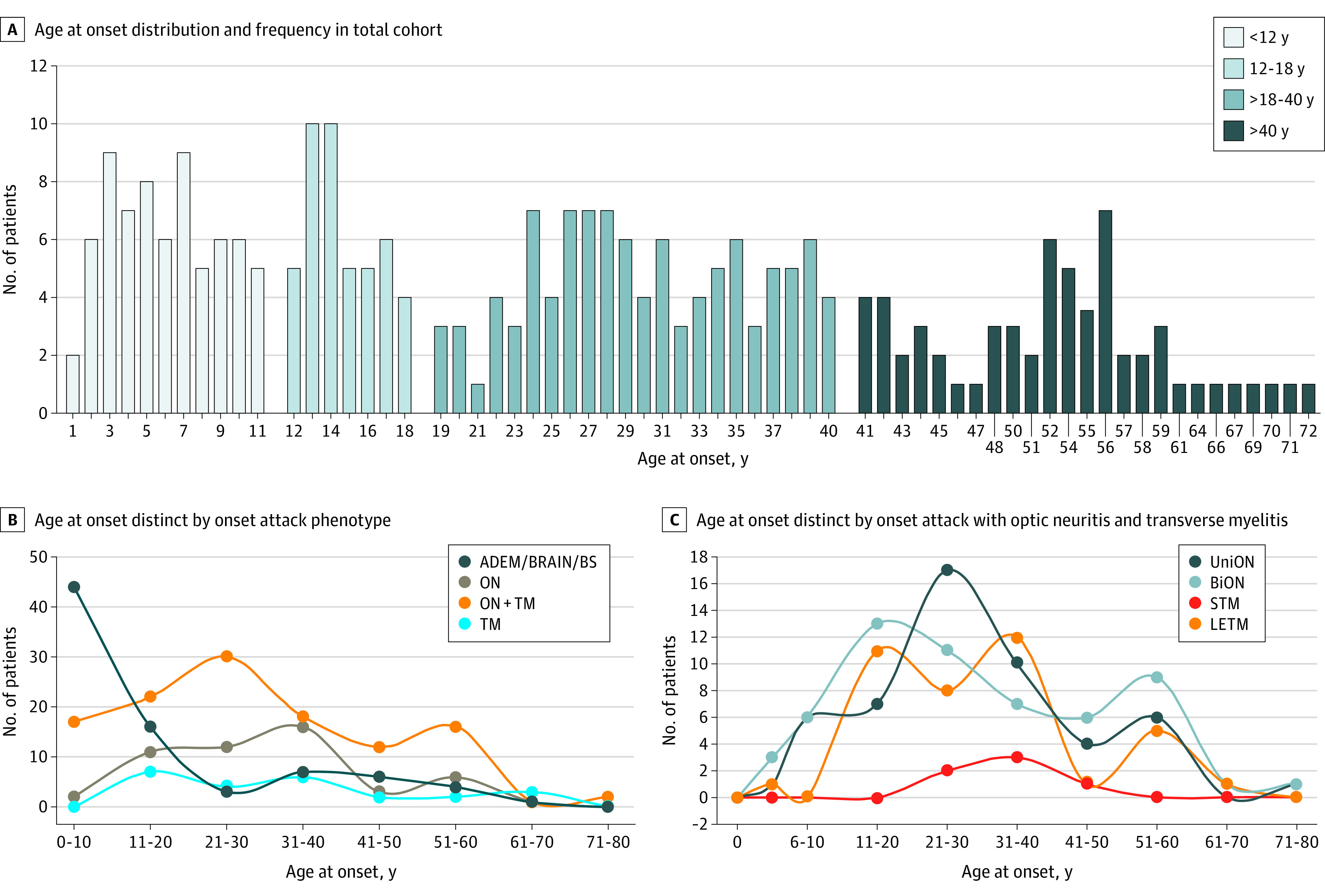
Age at Onset and Onset Attack Phenotype in Total Cohort

Onset age groups for subgroup analysis were divided into ages younger than 12 years, 12 to 18 years, older than 18 to 40 years, and older than 40 years, also referred to as young pediatric, teenage, young adult, and older adult groups, respectively. The age of 12 years was selected as being a reasonably pragmatic cutoff for puberty, and the age range of 12 to 18 years was chosen for teenage years; other groups were identified after reviewing the frequency plot (Figure 1A) to ensure that an equitable number of patients were represented in each group. To compare the association of immunosuppression and immunomodulation with relapse outcomes, we analyzed only patients with relapsing disease in treatment analysis (a first clinical event in MOGAD is not a currently established indication for long-term immunosuppression or immunomodulation). We categorized patients into 5 maintenance therapy subgroups: (1) prednisolone (P), (2) steroid-sparing immunosuppressive treatment (IST; ie, azathioprine, methotrexate, or mycophenolate mofetil), various combinations of 1 and 2, (3) MS-disease–modifying therapy (MS-DMT), (4) intravenous immunoglobulin (IVIG; alone or in combination with rituximab, P, IST, or P and IST), and (5) rituximab (alone or in combination with P, IST, or P and IST). The no-treatment group refers to the off-treatment phase among patients in the relapsing group, which could occur between any attack or between the last attack and last follow-up. First-line therapy was analyzed for treatment groups with sufficient numbers of patients. Patients who received treatment with cyclosporin, cyclophosphamide, tocilizumab, or mitoxantrone were excluded from the survival analysis because of the low number of patients. Annualized relapse rates (ARRs) were calculated as number of relapses per year (inclusive of incident event) and included patients with at least 12 months of follow-up to prevent overinflation of rates.

### Statistical Analysis

We applied χ^2^ and 1-way analysis of variance (or the Kruskal-Wallis test) to compare multiple groups of data between subgroups. Kaplan-Meier survival curves were used for the time-to-event analyses, and across-group comparisons used a univariate Cox proportional regression model. Univariate analyses were performed for nontreatment factors (ie, sex, race, health care center, onset age, and onset phenotype on relapse risk). These analyses were adjusted for other factors as indicated in the results. Analyses for maintenance therapies and risk of relapse were performed using multivariate models, adjusting for covariates. We selected covariates associated with a change in relapse risk (using a cutoff of *P* = <.05) from univariate analysis. Covariates were age at onset, presence of ON at onset, any TM at onset, and number of relapses prior to immunosuppression or immunomodulation therapy when not analyzing first-line therapy only. *P* values were 2 sided, and statistical significance was set at *P* ≤ .05. The study analyzed data from January 1973 to March 2020. Stata statistical software version 16.0 (StataCorp) packages were used for analysis, and data were analyzed from April through July 2020

## Results

### Patient Clinical Characteristics

Among 276 patients identified with MOGAD, 183 patients were categorized in the incident group. Median (IQR) follow-up was 24.4 (9.6-44.4) months, and 58 patients had 4 years of follow-up or more. Demographic data are shown in Table 1. In the total cohort, there were more female patients (166 [60.1%] female patients) and the mean (SD) onset age was 26.4 (17.6) years. In the incident group, there were 106 (57.9%) female patients, and the mean (SD) age at onset was 28.2 (18.1) years. The total median (range) age in our study was similar to that in a 2021 study by Cobo-Calvo et al^10^ (25 [1-72] years vs 29.9 [16.7-41.7] years) (Table 2).^9,10,11,12,19,20,23,24^ Among 246 patients for whom race was documented in the total cohort, there was no obvious racial bias: there were 28 Asian individuals (11.4%), 8 Black individuals (3.3%), 204 White individuals (79.7%), and 6 individuals with mixed or other race (2.4%), while 86% of the UK population is White according to Gov.UK 2020.^25^ Mixed race and other race were combined owing to low numbers; mixed race was a specific term used in data collection. There was an increased proportion of Asian individuals in the young pediatric group (12 of 57 individuals with race data [21.1%]) compared with other age subgroups.

**Table 1.** Demographic and Baseline Clinical Characteristics of Patients

Characteristic	Patients, No. (%)					
	Total cohort					Incident group total (n = 183)
	Total (N = 276)	<12 y (n = 69)	12-18 y (n = 45)	>18-40 y (n = 103)	>40 y (n = 59)	
Age at onset, y						
Mean (SD)	26.4 (17.6)	6 (2.9)	14.6 (1.9)	30 (5.9)	52.6 (8.2)	28.2 (18.1)
Median (range)	25 (1-72)	6 (1-11)	14 (12-18)	29 (19-40)	52 (41-72)	28 (1-72)
Sex						
Female	166 (60.1)	40 (58.0)	26 (57.8)	65 (63.1)	35 (59.3)	106 (57.9)
Male	109 (39.6)	29 (42.0)	19 (42.2)	38 (36.9)	24 (40.7)	77 (42.1)
Race						
No. with data	246	57	41	96	55	158
Asian total	28(11.4)	12 (21.1)	3 (7.9)	7 (7.3)	6 (10.9)	18 (11.4)
South Asian	24 (9.8)	10 (17.5)	3 (7.9)	7 (7.3)	4 (7.3)	14 (8.9)
East Asian	4 (1.6)	2 (3.5)	0	0	2 (3.6)	4 (2.5)
Black	8 (3.3)	2 (3.5)	0	2 (2.1)	4 (7.3)	8 (5.1)
White	204 (79.7)	43 (75.5)	33 (86.8)	84 (87.5)	44 (80)	131 (82.9)
Mixed race or other race^a^	6 (2.4)	0	2 (5.3)	3 (3.1)	1 (1.8)	1 (0.6)
Disease duration, mo						
Mean (SD)	62.1 (78.8)	81.9 (104.7)	73.7 (76.3)	58.6 (76.3)	42.7 (47.9)	33.1 (33.7)
Median (range)	35.0 (1.2-540.0)	44.0 (1.2-540.0)	50.7 (2.3-398.5)	33 (1.6-422.6)	29.7 (3.3-256)	24.4 (1.2-235.1)
Clinical attack at onset						
No. with data	275	69	45	102	59	183
ON	119 (43.3)	20 (29.0)	15 (33.3)	53 (52.0)	31 (52.5)	76 (41.5)
Unilateral, No.	52	7	6	28	11	31
Bilateral, No.	58	12	9	20	17	38
Unidentified, No.	9	1	0	5	3	6
TM	51 (18.5)	2 (2.9)	10 (22.2)	29 (28.4)	10 (16.9	37 (20.2)
STM, No.	6	0	0	5	1	4
LETM, No.	39	1	10	21	7	31
Unidentified length TM, No.	6	1	0	3	2	2
ON with TM	24 (8.7)	0	7 (15.6)	10 (9.8)	7 (11.9)	18 (9.8)
ADEM, brain, or BS	81 (29.5)	47 (68.1)	13 (28.9)	10 (9.8)	11 (18.6)	52 (28.5)
Follow-on steroid after onset attack						
No. with data	230	47	38	90	55	135
None	79 (34.4)	9 (19.2)	15 (39.5)	38 (42.2)	17 (30.9)	39 (25.2)
<2 mo	39 (16.9)	16 (34.0)	8 (21.1)	11 (12.2)	4 (7.3)	27 (17.4)
2-6 mo	54 (23.5)	18 (38.3)	11 (28.9)	18 (20.0)	7 (12.7)	17 (23.9)
>6 mo	58 (25.2)	4 (8.5)	4 (10.5)	23 (25.6)	27 (49.1)	52 (33.5)
Disease course						
Monophasic	137 (49.6)	32 (46.4)	21 (45.1)	48 (47.4)	36 (61.0)	137 (74.9)
Relapsing	139 (50.4)	37 (53.6)	24 (54.9)	55 (52.6)	23 (39.0)	46 (25.1)
Unmatched CSF oligoclonal band						
No. tested	165	32	33	64	36	96
With outcome	20 (12.1)	7 (21.8)	1 (3.0)	7 (10.9)	5 (13.8)	15 (15.6)
Family history of other autoimmune diseases						
No. with data	205	60	32	66	47	139
With outcome	68 (33.2)	8 (3.3)	8 (25.0)	33 (50.0)	19 (40.4)	43 (30.9)
Associated with other autoantibodies						
No. with data	268	68	43	101	56	176
With outcome	32 (11.9)	7 (10.3)	10.2 (49.0	14 (13.9)	6 (10.7)	20 (11.4)
ARR, mean (SD)^b^	0.59 (0.50	0.56 (0.60)	0.57 (0.38)	0.62 (0.46)	0.60 (0.53)	0.58 (0.49)

Abbreviations: ADEM, acute disseminated encephalomyelitis; ARR, annual relapse rate; BS, brain stem; CSF, cerebrospinal fluid; LETM, long extensive transverse myelitis; ON, optic neuritis; STM, short transverse myelitis; TM, transverse myelitis.

^a^ Individuals with mixed race and other race were those who self-identified as others outside of the categories listed in this table. Mixed race and other race were combined owing to low numbers; mixed race was a specific term used in data collection.

^b^ For ARR, only individuals with disease duration of 12 months or more were included.

**Table 2.** Comparison With Previous Studies

Study characteristic	Satukijchai et al		Jurynczyk et al, 2017^11^		Cobo-Calvo et al, 2018^23^		Cobo-Calvo et al, 2021^10^	Jarius et al, 2016^19^	Pandit et al, 2018^24^	Cobo-Calvo et al, 2019^9^	Senanayaka et al, 2019^12^	Ramanathan et al, 2018^20^
	Total cohort	Incident group	Total cohort	Incident group	Total cohort	Incident group						
Country	UK	UK	UK	UK (Oxford)	France	France	France	German	India	France and Spain	Sri Lanka	Australia and New Zealand
Center	Multicenter	Multicenter	Multicenter	Single center	Multicenter	Multicenter	Multicenter	Multicenter	Single center	Multicenter	Multicenter	Multicenter
Patients, No.	276	204	252	44	197	139	366	50	42	125	126	59
Female patients (%)	60.3	57.6	57.0	48.0	49.2	47.48	51.0	74.0	42.9	55.2	56.0	67.8
Age at onset, median (range), y	25 (1-72)	28 (1-72)	Mean (SD), 30.1 (18.3)	Mean (SD), 32.0 (17.6)	36.5 (19.0-76.8)	37.4 (18.9-76.7)	29.9 (16.7-41.7)	31 (6-70)	21 (6-53)	34.1 (18.0-67.1)	26 (3-68)	12 (1-74)
Children (No.)	114 (≤18 y)	68 (≤18 y)	81 (≤16 y)	NA	All adult	All adult	98 (<18 y)	8 (<18 y)	13 (<18 y)	All adult	44 (<18 y)	33 (≤16 y)
Predominate racial or ethnic group (%)	White (79.7)	White (71.7)	White (78.2)	NA	White (92.9)	White (91.4)	NA	White (98.0)	Asian (100)	White (96.0)	Sinhalese (85.0)	White (72.9)
Disease duration, median (range), mo	36.0 (1.2-540.0)	24.0 (1.2-235.1)	26.0 (0-492.0)	15.5 (1.0-57.0)	15.8 (1.0-556.0) FU duration	10.8 (1.0-37.0) FU duration	36.0 (6.0-637.0)	Monophasic: 26.0 mo; relapsing: 52.5 mo FU duration	57.6 (12.0-336.0)	54.0 (2.4-564.0) FU duration	48.0 (1.4-240.0) FU duration	45.0 (12.0-288.0) FU duration
Disease course (% relapsing)	50.4	37.1	44.0	36.0	42.1	26.6	55.2	80.0	57.1	Only relapse	34.0	Only relapse
ARR, mean (SD)	0.59 (0.45)	0.66 (0.52)	0.2 (Oxford incident group)	NA	0.37 (0.79)	0.38 (0.92)	0.23 (0.35)	Median (range), 0.83 (0.05-6.92)	1.04 (0.03)	0.79 (0.91)	NA	NA
Onset phenotype (%)	ON (43.3)	ON (41.5)	Unilateral ON (31.0)	Unilateral ON (18.0)	ON (60.9)	ON (64.1)	ON (40.8)	ON (64.0)	ON (40.5)	ON (65.6)	ON (51.0)	ON (54.0)
			Bilateral ON (24.0)	Bilateral ON (27.0)								
	TM (18.5)	TM (20.2)	TM (18.0)	TM (21.0)	TM (22.3)	TM (17.9)	TM (12.2)	TM (18.0)	TM (52.4)	TM (20.0)	TM (23.0)	ADEM (20.0)
	ON with TM (8.7)	ON with TM (9.8)	ON with TM (9.0)	ON with TM (9.0)	ON with TM (7.6)	ON with TM (8.6)	ON with TM (4.1)	ON with TM (10.0)	BS (2.4)	ON with TM (7.2)	ON with TM (6.0)	Other (26.0)
	ADEM, brain, BS (29.5)	ADEM, brain, BS (28.5)	ADEM, ADEM-like (18.0)	ADEM, ADEM-like (25.0)	BS (4.0)	BS (2.9)	ADEM (36.7)	BS (2.0)	Encephalitis (4.7)	BS with encephalitis (7.2)	ADEM (9.0)	NA
					BS with encephalitis (2.0)	BS with encephalitis (2.2)						
	NA	NA	NA	NA	BS with ON (0.5)	BS with ON (0.7)	BS (6.1)	Encephalitis with myelitis (6.0)	NA	NA	ON with intractable hiccup (1.0)	NA
					Encephalitis (2.5)	Encephalitis (3.6)						
	NA	NA	NA	NA	NA	NA	NA	NA	NA	NA	ON with ADEM (10.0)	NA
MOG assay	Live CBA a1	Live CBA a1	Live CBA a1	Live CBA a1	Live CBA a1	Live CBA a1	Live CBA a1	Live CBA a1	Live CBA a1	Live CBA a1	Live Flow a1	Live flow a1
MOG construct secondary Ab	MOG IgG1	MOG IgG1	MOG IgG1	MOG IgG1	MOG-GFP IgG	MOG-GFP IgG	MOG-GFP IgG	MOG-GFP IgG	MOG IgG	MOG IgG	MOG IgG1	MOG-GFP IgG

Abbreviations: Ab, antibody; ADEM, acute disseminated encephalomyelitis; BS, brain stem; CBA, cell-based assay; FU duration, follow-up duration; GFP, green fluorescent protein; NA, not applicable; ON, optic neuritis; ON with TM, simultaneous optic neuritis with transverse myelitis; TM, transverse myelitis.

As expected, the incident group had a shorter median disease duration and an increased proportion of patients with monophasic disease. The age of onset had 2 peaks in childhood (below and above age 12 years), 1 peak between ages 19 and 40 years, and another peak between ages 50 and 60 years (Figure 1A). There were similar sex ratios, rates of coexisting other autoantibodies, and annual relapse rates across age groups (Table 1). Although the 2 younger age of onset groups had the longest disease duration, 37 of 69 patients (53.6%) in the youngest age group had relapsing disease, which is similar to rate of the older age groups. Oligoclonal bands were more frequent in the young pediatric group and less frequent in the teenage group (Table 1).

### Clinical Onset Presentation

Among 275 patients with presentation data, ON was the most common presentation (119 patients [43.3%]), with approximately equal proportions of these patients having unilateral involvement (52 patients [47.3%]) and bilateral involvement (58 patients [52.7%]). Fewer than 10% of patients with presentation data presented with simultaneous ON and TM (24 patients [8.7%]). Similar distributions were seen in the incident group (Table 1). Among 69 patients in the young pediatric group, more patients presented with ADEM, brain, or brainstem attacks (47 patients [68.1%]), fewer patients presented with transverse myelitis (2 patients [2.9%]), and fewer patients (4 patients [8.5%]) were given follow-on prednisolone (ie, >6 months) after acute therapy (Table 1).

The associations between onset age and clinical onset phenotype distributions are shown in Figure 1B and C. There were 2 peaks for ON, at approximately ages 21 to 30 years and 51 to 60 years, with similar distributions for unilateral and bilateral involvement. TM with or without ON was uncommon in childhood, while long extensive transverse myelitis was most likely to present among adults aged between 20 and 40 years and a short TM peak was observed at approximately age 40 years (Figure 1B and C).

Relapse clinical phenotypes at last follow-up among 139 patients with relapses subdivided into 4 age groups (eFigure in the Supplement). The most common overall phenotypes were relapsing ON (39 patients [28.1%]) or relapsing ON with TM (ie, patients who had relapsing ON and TM [including short or long extensive TM] sequentially in any order or simultaneously; 40 patients [28.8%]). However, among 38 patients aged younger than 12 years, ADEM followed by ON was the most common phenotype (7 patients [18.4%]). Relapsing TM was found only among adults (ie, those, aged >18 years), and relapsing ADEM was found only among patients aged younger than 12 years (eFigure in the Supplement).

### Risk of Relapse After Onset Attack

**Figure 2.**
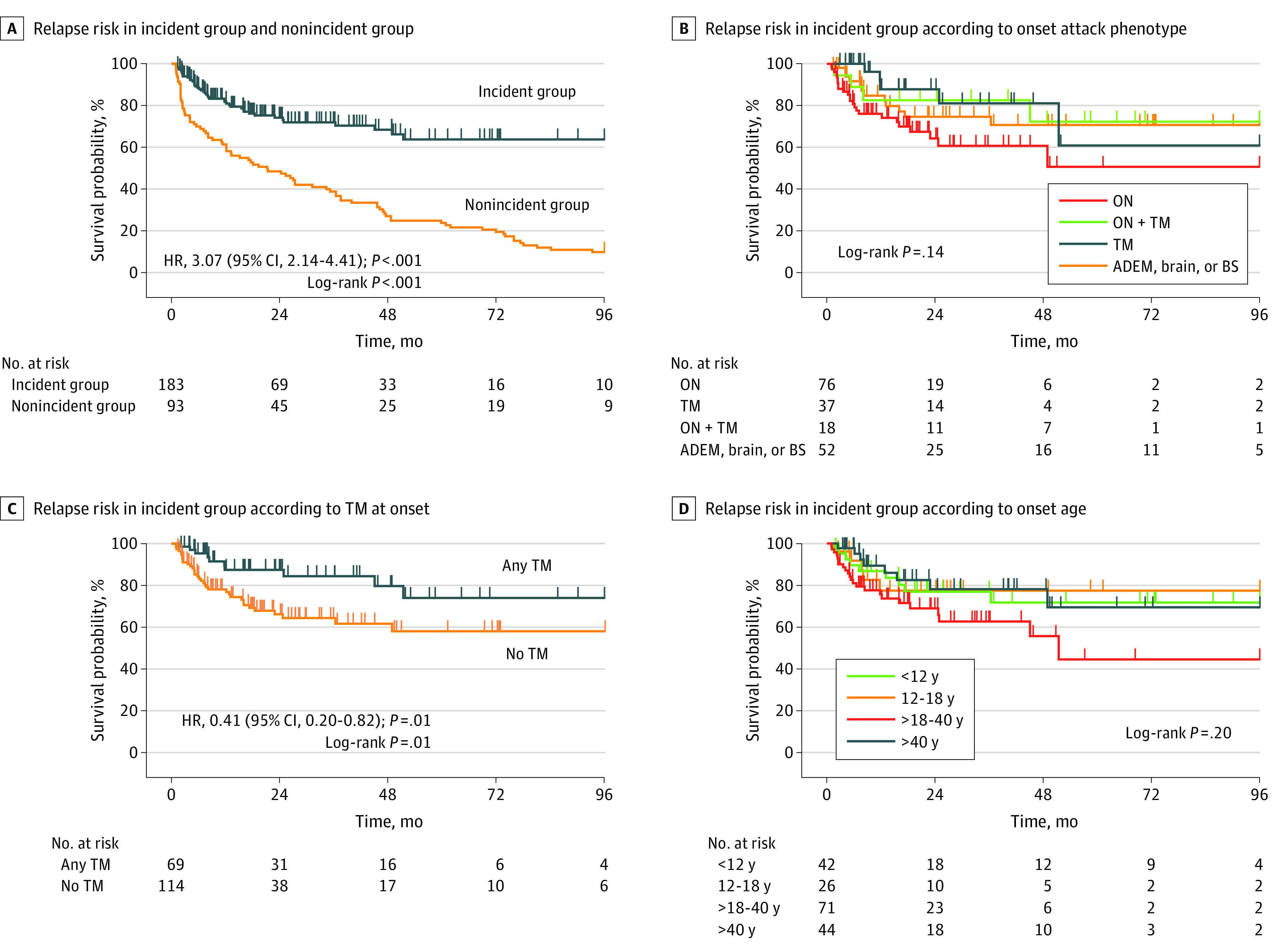
Relapse Risk in Incident and Nonincident Groups

Because the MOG antibody test has been available only in recent years, patients with onset many years ago will be unlikely to represent for diagnosis unless they relapse. Thus, relapse risk among individuals with onset long ago is likely to appear higher that its true value, and this will be associated with a bias toward an increased relapse rate in the nonincident group. Figure 2A demonstrates this by showing a significantly increased risk of relapse in the nonincident group compared with the incident group (hazard ratio [HR], 3.07; 95% CI, 2.14-4.41; *P* < .001), with a 73.1% (95% CI, 63.9%-81.7%) risk over 4 years. In the incident group, 4-year risk of relapse was 31.7% (95% CI, 23.9%-41.2%) and 8-year risk of relapse was 36.3% (95% CI, 27.1%-47.5%). Sex, race, and study site (adjusted for age) were not associated with risk of relapse (eTable 2 in the Supplement).

**Figure 3.**
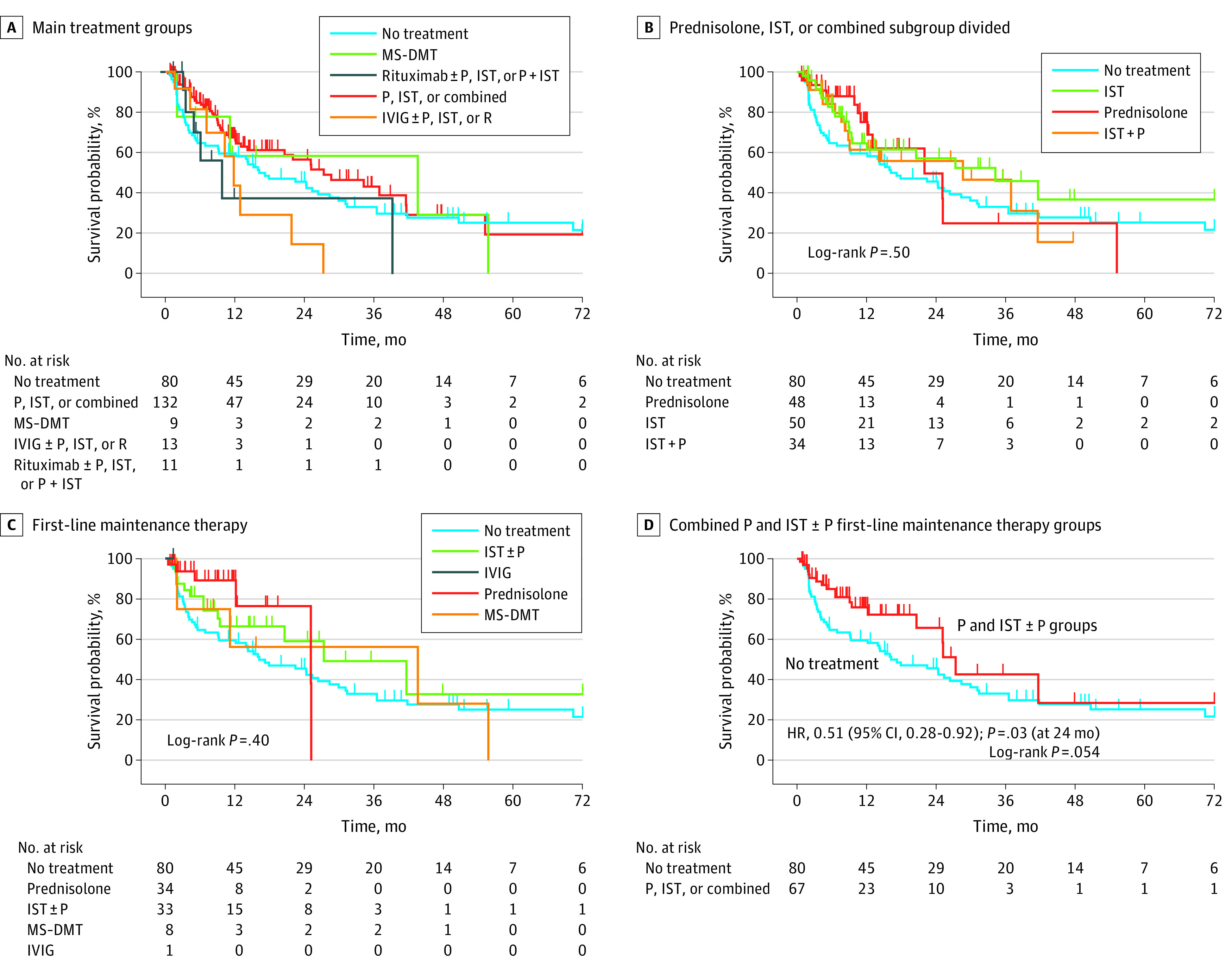
Relapse Risk in Total Cohort

Figure 2B shows risk of relapse in 4 main onset phenotypes, with no overall association. In intragroup comparison, the ON onset group had a significantly increased risk of relapse compared with the TM onset group (HR, 2.66; 95% CI 1.01-6.98; *P* = .047). However, because fewer patients with ON at onset than patients with TM at onset received a follow-on course of oral prednisolone, this treatment was added as a covariate, and there was no significant difference in risk of relapse (HR, 2.37; 95% CI, 0.79-7.01); *P* = .12). Patients with TM at onset alone or in combination (ie, any TM) had a significantly decreased risk of relapse compared with those without TM (HR, 0.41; 95% CI, 0.20-0.82; *P* = .01) (Figure 3C), and this association remained when a follow-on course of prednisolone was included as a covariate (HR, 0.47; 95% CI, 0.22-0.99; *P* = .049).

There was no linear association of onset age with time to relapse (Figure 2D); however, because the onset phenotypes any TM at onset and isolated ON at onset were associated with risk of relapse, there was a significantly increased risk of relapse for adults aged older than 18 years to 40 years compared with those aged older than 40 years when adjusting for any TM at onset and isolated ON at onset (HR, 2.71; 95% CI, 1.18-6.19; *P* = .02). The 4-year risk of relapse was 29.3% (95% CI, 15.8%-50.1%) for the under age 12 years group, 23.7% (95% CI, 10.5%-48.1%) for the ages 12 to 18 years group, 45.8% (95% CI, 29.4%-65.9%) for the ages older than 18 years to 40 years group, and 22.5% (95% CI, 11.2%-42.4%) for the ages older than 40 years group. The 8-year risk of relapse was 29.3% (95% CI, 15.8%-50.1%) for the under age 12 years group, 23.7% (95% CI, 10.5%-48.2%) for the ages 12 to 18 years group, 59.4% (95% CI, 35.1%-84.8%) for the ages older than 18 years to 40 years group, and 32.3% (95% CI, 15.4%-59.5%) for the ages older than 40 years group.

### Association of Maintenance Immunosuppression With Relapsing MOGAD

There were 139 patients with relapses, among whom 131 patients had sufficient treatment data for inclusion in analysis. Of these individuals, 100 patients (76.3%) were started on immunosuppression or immunomoderation at some point to prevent long-term relapse. Moreover, 32 patients started on treatment (32.0%) switched their treatment strategy, including 17 patients who did so owing to lack of therapeutic efficacy. Most patients started on treatment (76 individuals [76.0%]) received first-line maintenance therapy after the first relapse; of those patients, 34 individuals (44.7%) were treated with prednisolone alone, 33 patients (43.4%) were treated with conventional noncorticosteroid IST (alone or in combination with prednisolone), 8 patients (10.5%) received MS-DMTs (glatiramer acetate for 1 patient, interferon beta for 5 patients, and dimethyl fumarate for 2 patients), and 1 patient (1.3%) received IVIG. There were 7 patients who received cyclosporin, cyclophosphamide, tocilizumab, or mitoxantrone during the course of treatment, but none received these treatments as first-line therapy. When including treatment switches, there were 245 different treatment periods among these patients, including no-treatment periods. Indication biases may be associated with the chosen treatment; for example, rituximab and IVIG were given later in the disease course (except for 1 child aged 8 years who received IVIG as first-line therapy). Thus, we adjusted for number of relapses prior to treatment, along with factors associated with relapse risk: onset age, presence of isolated ON at onset, and presence of any TM at onset in a multivariate analysis (eTable 3 in the Supplement).

No overall significant difference was found in time to relapse across 5 main treatment groups, although numbers in some groups were small (Figure 3A). Differences in risk of relapse among patients treated with prednisolone, IST, or prednisolone with IST compared with the no-treatment group were not statistically significant (HR, 0.65; 95% CI, 0.42-1.01; *P* = .06). There was no difference in risk of relapse after adjustment for previously identified covariates. The group treated with rituximab relapsed sooner, with an initial decline in the MS-DMTs survival curve (Figure 3A). Figure 3B shows the separation of the most common treatment group to explore if there was any differences among prednisolone alone, IST alone, and a combination (IST with P). There was no significant difference across the groups as a whole; however, median (IQR) time to relapse was shorter in the no-treatment group (16.2 [3.4-70.4] months) compared with the treatment groups (prednisolone: 22 [12.2-25.2] months; IST: 34.3 [8.9-108.5] months; IST with P: 28.7 [8.3-41.6] months).

It is difficult to fully adjust for indication bias; thus, we compared first-line maintenance therapies with the no-treatment group using multivariate analysis, including age at onset, isolated ON at onset, and any TM at onset as covariates (Figure 3C). Although across all groups there was no significant difference, in multivariate analysis at 24 months, there was a significantly decreased risk of relapse for patients in the prednisolone group compared with patients in the no-treatment group (HR, 0.33; 95% CI, 0.12-0.92; *P* = .03) (Figure 3C). The cutoff of 24 months was considered because no patient took prednisolone longer than 25.2 months. The risk of relapse in 2 years was 54.4% (95% CI, 43.7%-65.9%) for the no-treatment group compared with 23.5% (95% CI, 7.5%-60.1%) for the prednisolone group, 40.9% (95% CI, 23.9%-63.9%) for the IST with or without P group, and 43.8% (95% CI, 15.9%-85.3%) for the MS-DMT group. Moreover, when combining main first line therapies (ie, prednisolone alone and IST with or without prednisolone groups combined), there was a significantly decreased risk of relapse compared with the no-treatment group when adjusting for covariates at 24 months before the numbers become too small to analyze (HR, 0.51; 95% CI, 0.28-0.92; *P* = .03) (Figure 3D).

## Discussion

This cohort study was a multicenter collaboration analyzing UK patients with MOGAD with good representation from all age groups, which included a large incident group with longer follow-up than seen in previous studies. Overall, we found a 31.7% risk of relapse within 4 years and a 36.3% risk of relapse within 8 years; risk was increased among young adults (ie, those aged older than 18 to 40 years), with a 45.8% risk of relapse within 4 years and a 59.4% risk within 8 years. Having ON alone at onset was associated with increased risk of relapse before adjusting for a course of oral prednisolone, and any TM was associated with decreased risk. Proportions of the population by race varied by age group, with an increased proportion of Asian individuals in the young pediatric group. There were also more ADEM presentations in the young pediatric group. First-line therapy with prednisolone, nonsteroidal immunosuppressants (ie, azathioprine, mycophenolate, or methotrexate), or a combination was associated with decreased risk of relapse by approximately 50%.

This study supports baseline features across ages noted in previous large MOGAD cohort reports.^9,10,11,12,19,20,23,24^ A 2021 large MOGAD cohort publication^10,15^ compared baseline features across age ranges that are similar to those investigated in our study and noted more ADEM at onset and less ON at onset in the young pediatric group but not in the older pediatric or adolescent group, which is in line with our findings. We did not find a decreased percentage of TM in the adolescent group, although our age categorization was slightly older: ages 12 to 18 years compared with 10 to 17 years in the Cobo-Calvo et al^10^ cohort. In accordance with their study, we also noted an increased relapse risk in the young adult group. However, unlike our study, Cobo-Calvo et al^10^ did not find decreased risk with any TM, but the cohorts differed in that our analysis was performed on an incident group (ie, focused on individuals diagnosed at onset). The findings of a previous study^11^ suggested that a course of oral prednisolone after the onset attack for more than 3 months were associated with decreased risk of relapse, and we noted that patients with ON at onset were less likely to receive a tapering off of oral prednisolone. This may be associated with such patients being seen first by ophthalmologists, who in the UK may be influenced by the Beck study.^26^ That study’s findings suggested that oral prednisolone may increase the risk of relapse after an attack of ON. Adjusting for a tapering off of prednisolone (categorized as any prednisolone or a longer course) removed the ON alone vs TM alone effect but not the decreased risk of relapse among individuals with any TM at onset.

In a mixed cohort, patients experiencing relapse with transverse myelitis may be more likely to be referred than patients experiencing relapse with optic neuritis, which could obscure differences in the results. Additionally, we noted variation in racial proportions across age groups, which was not previously studied, to our knowledge. Moreover, a lack of short TM and relapsing TM has not been seen in pediatric groups and relapsing ADEM has not been seen in adults, to our knowledge. It is interesting to note that, in our study, bilateral ON was predominant except among young adults.

Only an incident study can give an unbiased cohort risk of relapse over time. There have been 2 previous incident studies reported, one with 44 patients included in our study with a shorter follow-up (median 15.5 months)^11^ and one from the Cobo-Calvo et al study^23^ of 139 adult patients with a shorter follow-up time (median, 10.78 months). These studies reported a 27% to 29% risk of relapse over 11 to 12 months and a 40% to 43% risk at 24 months. Our longer follow-up time (median [IQR], 24.4 [9.6-44.4] months) and larger incident group (183 patients) had 58 patients with at least a 4-year follow-up, and we found a 4-year relapse risk of 31.7%. The decreased rate in our study is likely associated with the inclusion of more children, who have a decreased relapse risk compared with young adults. Additionally, in the UK, some patients are treated with low-dose prednisolone for 3 to 12 months after onset attack to decrease risk of early rebound or relapse. This suggests that when extrapolating risk of relapse from studies, early treatment practice and the age of the patient need to be considered.

Reports suggesting the effectiveness of corticosteroids and immunosuppression in relapse prevention in smaller groups^11,19,20^ were more convincing in a larger study using propensity score matching. The study found that the long-term immunosuppression or immunomodulation treatment group (40 patients treated with azathioprine, mycophenolate, or rituximab) had a decreased risk of relapse compared with 59 patients receiving no treatment, with an HR of 0.41; however, corticosteroid treatment was not included owing to the low number of patients.^9^ We found that using prednisolone, IST, or a combination as first-line treatment was associated with a similar decrease in relapse risk, with an HR of 0.51 vs no treatment.

### Limitations and Strengths

Our study has some limitations. It included a small number of patients in some of subgroups, and multiple treatments were combined for some patients, making it difficult to separate associations of individual treatments and adjust for indication bias. However, including covariates that may bias relapse risk and using only first-line treatments should help adjust for these biases. Only time to next relapse was analyzed in the study, which reflects the clinical labeling of patients with monophasic or relapsing phenotypes and also the primary outcome in clinical trials. However, future, larger studies should include more complex recurrent event analyses to assess the association of treatment with subsequent relapses. It is important to consider caution in the interpretation of findings when there are multiple interactions and confounders and small numbers of patients, which is why we tested covariates based on factors previously reported to be associated with relapse risk or based on results in this study.

The strengths of our study are that it includes the largest and longest follow-up incident group of patients MOGAD, to our knowledge. We were also able to study differences across a range of age categories separating prepubertal children from adolescents and young adults from older adults in order to investigate relapse risk by age. Additionally, we were able to study the association of prednisolone with relapse prevention, which is a common treatment used in relapsing MOGAD.

## Conclusions

We found that MOGAD was associated with distinct clinical presentations, demographics, and relapse risks across age categories with a decreased risk of relapse overall than previously reported. Our study found that young adults had an increased risk of relapse and that standard first-line immunosuppression or prednisolone were associated with a decrease in the risk of relapse in relapsing patients by nearly one-half. Understanding longer-term relapse risk may help clinicians make a more informed decisions about when to start immunosuppression treatment considering its association with milder disability from relapses compared with AQP4 antibody NMOSD. Currently, the evidence for maintenance therapies for relapse prevention in MOGAD is poor and relies on observational studies, and therefore future prospective randomized clinical trials are crucial to fill this gap.

## Notes

Supplement. **eFigure.** Clinical Phenotype in Relapsing Disease by Age Group **eTable 1.** Clinical Presentation at Onset in ADEM, Brain, and BS Subgroup **eTable 2.** Risk of Relapse by for Sex, Race, and Study Site in Total Cohort **eTable 3.** Covariates Included in Treatment Analysis
