# Errors in Table and Abstract

**DOI:** 10.1001/jamanetworkopen.2022.5056

**Published:** 2022-03-09

In the Original Investigation titled “Factors Associated With Relapse and Treatment of Myelin Oligodendrocyte Glycoprotein Antibody–Associated Disease in the United Kingdom,”^1^ published January 10, 2022, there were data errors in the columns displaying results for the total cohort and incidence group under the header “Satukijchai et al” in Table 2 and an error in a 95% CI given in the Abstract for the association between transverse myelitis at onset and risk of relapse. This article has been corrected.^1^
