# Supplementary material for: Factors Associated With Relapse and Treatment of Myelin Oligodendrocyte Glycoprotein Antibody–Associated Disease in the United Kingdom
Source: JAMA Netw Open. 2022 Jan 10;5(1):e2142780. doi: 10.1001/jamanetworkopen.2021.42780 (PMC8749481; doi:10.1001/jamanetworkopen.2021.42780)
Supplement: Supplement. — eFigure. Clinical Phenotype in Relapsing Disease by Age Group eTable 1. Clinical Presentation at Onset in ADEM, Brain, and BS Subgroup eTable 2. Risk of Relapse by for Sex, Race, and Study Site in Total Cohort eTable 3. Covariates Included in Treatment Analysis [file jamanetwopen-e2142780-s001.pdf]

Supplemental Online Content

Satukijchai C, Mariano R, Messina S, et al. Factors associated with relapse and treatment of myelin oligodendrocyte glycoprotein antibody–associated disease in the United Kingdom. *JAMA Netw Open*. 2022;5(1):e2142780.
doi:10.1001/jamanetworkopen.2021.42780

eFigure. Clinical Phenotype in Relapsing Disease by Age Group
eTable 1. Clinical Presentation at Onset in ADEM, Brain, and BS Subgroup
eTable 2. Risk of Relapse by for Sex, Race, and Study Site in Total Cohort
eTable 3. Covariates Included in Treatment Analysis

This supplemental material has been provided by the authors to give readers additional information about their work.

© 2022 Satukijchai C et al. *JAMA Network Open*.

eFigure. Clinical Phenotype in Relapsing Disease by Age Group

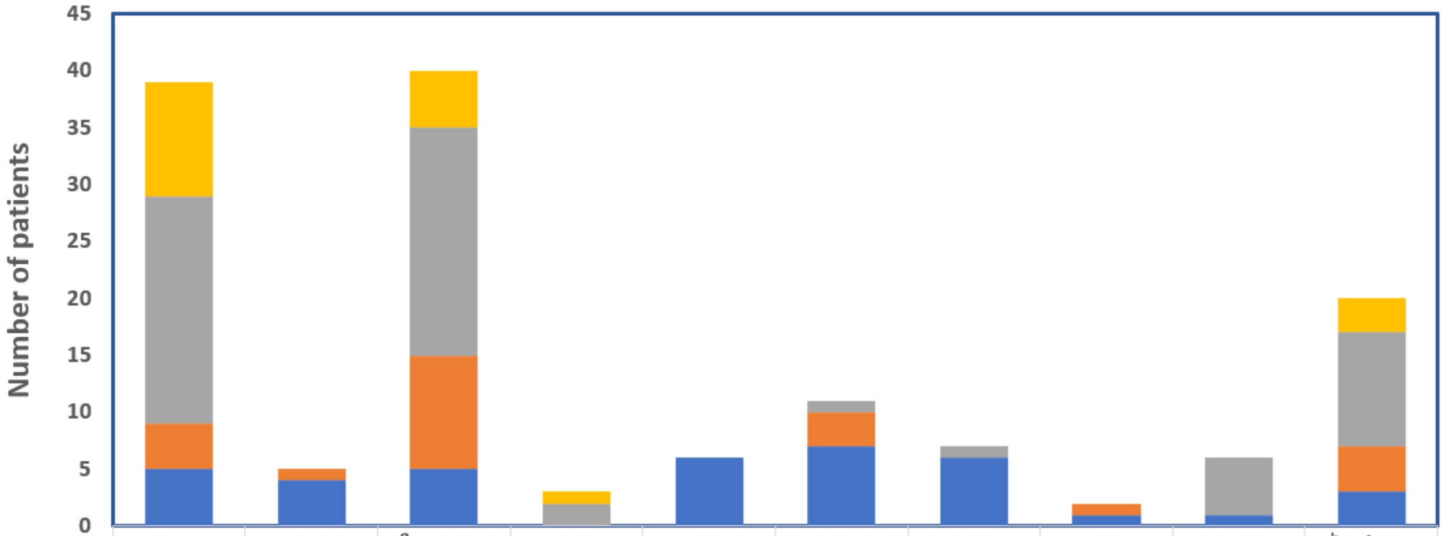

	ON	ON- BS	[a]ON+TM	TM	ADEM	ADEM- ON	ADEM -BS	BS	BS-ON	[b]Others
>40	10	0	5	1	0	0	0	0	0	3
>18-40	20	0	20	2	0	1	1	0	5	10
12-18	4	1	10	0	0	3	0	1	0	4
<12	5	4	5	0	6	7	6	1	1	3

Relapsing phenotype

Abbreviations: ON, optic neuritis; TM, transverse myelitis; ADEM, acute disseminated encephalomyelitis; BS, Brainstem

[a]ON+TM either sequentially in any order or simultaneously

[b]Those who have 3 or more phenotypes during the course of their disease

© 2022 Satukijchai C et al. *JAMA Network Open*.

eTable 1. Clinical Presentation at Onset in ADEM, Brain, and BS Subgroup

	Total cohort					Incident cohort
ADEM/Brain/BS	Total	<12 Years	12-18 Years	>18-40 Years	>40 Years	Total
	(n=81)	(n=47)	(n=13)	(n=10)	(n=11)	(n=52)
ADEM, n(%)	55(62.5)	41(87.2)	9(69.2)	3(30)	2(18.2)	39(75)
ADEM alone, n	43	32	8	2	1	31
ADEM with [a]other presentations, n	12	9	1	1	1	8
Brain without encephalopathy, n(%)	10(12.3)	5(10.6)	2(15.4)	3(30)	0	6(11.5)
Brain without encephalopathy alone, n	6	3	1	2	0	3
Brain without encephalopathy with [a]other presentations, n	4	2	1	1	0	3
Brain stem, n(%)	14(17.2)	1(2.1)	1(7.7)	3(30)	9(81.8)	6(11.5)
Brain stem alone, n	8	1	1	1	5	3
Brainstem with [a]other presentations, n	6	0	0	2	4	3
Cortical, n(%)	2(3.6)	0	0	1(10)	1	2(3.8)
Cortical with simultaneous ON and TM, n	1	0	0	0	1	1
Cortical with meningeal involvement , n	1	0	0	1	0	1

Abbreviations: ADEM, acute disseminated encephalomyelitis, BS; Brainstem
[a]other presentations; those including optic neuritis and or transverse myelitis

© 2022 Satukijchai C et al. *JAMA Network Open*.

eTable 2. Risk of Relapse by for Sex, Race, and Study Site in Total Cohort

**Clinical Characteristic**	**N**	**Log rank, P- value**	**Cox proportional hazards regression, HR (95%CI), *P*-value**
Sex		0.25	**Unadjusted**
Female	116		Reference
Male	110		0.81 (0.58-1.16), 0.26
Race		0.10	**Unadjusted**
Asian total	28		Reference
Black	8		1.55 (0.55-4.35), 0.41
White	204		0.76 (0.42-1.36), 0.35
[a]Mixed race or other	6		1.83 (0.65-5.13), 0.25
Study site		0.32	**Adjusted with age at onset**
Oxford and outreach sites	146		Reference
Liverpool	65		0.79 (0.53-1.17), 0.24
Birmingham Children's Hospital	32		0.71 (0.39-1.30), 0.27
Evelina Children's Hospital London	22		0.47 (0.20-1.12), 0.09
Cardiff Wales	11		0.46 (0.14-1.44), 0.18

[a]Individuals with mixed race and other race were those who self-identified as others outside of the categories listed in this table.

© 2022 Satukijchai C et al. *JAMA Network Open*.

eTable 3. Covariates Included in Treatment Analysis

Clinical Characteristic		N	Cox proportional hazards regression, HR (95%CI), *P*-value		
			**Unadjusted**	**Adjusted**	**Risk of relapse**
Phenotype at onset					
	ON	76	2.66(1.01-6.98), 0.047	2.37(0.8-7), 0.120[a]	↑
	TM	37	Reference	Reference[a]	
Phenotype at onset					
	No TM	69	Reference	Reference[a]	
	Any TM	114	0.41 (0.20-0.82), 0.012	0.47 (0.22-1.0), 0.049[a]	↓
Age at onset					
	>18-40	103	2.10(0.94-4.69), 0.072	2.71 (1.18-6.19), 0.018[b]	↑
	>40	59	Reference	Reference[b]	
Number of relapses prior to IS therapy		Significant different among treatment group, *P*< .001			

Abbreviations: ON; Optic neuritis, TM; Transverse myelitis, IS; immunosuppression/immunomodulation
[a]Adjusted covariate for phenotype at onset; follow on oral prednisolone
[b]Adjusted covariate for age at onset; any TM at onset and Isolated ON at onset

© 2022 Satukijchai C et al. *JAMA Network Open*.
